# Response to letter: ‘Towards equitable implementation of AI‐assisted screening for diabetic retinopathy: Perspectives on digital literacy, trust and system readiness in developing countries’

**DOI:** 10.1111/dme.70345

**Published:** 2026-05-19

**Authors:** Umar A. R. Chaudhry, Lakshmi Chandrasekaran, Alicja R. Rudnicka, On Behalf the ARIAS Research Group

**Affiliations:** ^1^ Department of Population Health & Policy, School of Health & Medical Sciences City St George's, University of London London UK

We read with interest the letter from Putra and Rohmiati, providing their perspectives on the role of AI‐assisted screening for diabetic retinopathy. We respond to each of their points as follows:

- This study^1^ was conducted in England, and respondents were from socio‐demographic population groups across different ages, sexes and ethnicities. We acknowledge that there are differences in perspectives for people living with diabetes and healthcare professionals based in different countries, particularly those from low‐ and middle‐income countries, and the study findings may not necessarily translate outside of the English NHS landscape. However, we do appreciate that in general, health literacy and a lack of understanding could impact ‘adoption of digital technology’.
- We read with interest regarding the importance of ‘algorithmic fairness’, and that such systems are often developed using population datasets, which might affect their applicability to certain ethnic groups. This was specifically addressed in a recent publication^2^ whereby automated retinal image analysis systems were evaluated for their sensitivity for diabetic retinopathy and demonstrated equitable performance across population sub‐groups, specifically, also for ethnicity.
- We agree that there were differing views from healthcare professionals in terms of AI‐assisted technologies affecting their roles and responsibilities. As mentioned previously, this study was based within the NHS in England, and there may be similar or differing views depending on the context of healthcare systems and models.
- Similarly, it is difficult to comment on the level of trust (in terms of acceptability and engagement of AI systems) and the impact of institutional trust within healthcare systems across different countries from people living with diabetes, as this study was conducted only in England.
- The future suggested work specified in the study^1^ was to focus on outreach educational resources to address potential barriers/facilitators of AI acceptance in the English NHS Diabetic Eye Screening Programme. It would be interesting to identify any similarities or differences if this work were to be conducted in countries with different socio‐demographic profiles, levels of health literacy and healthcare systems.

We are grateful for the comments shared by Putra and Rohmiati and providing a discussion about the implementation of AI‐assisted diabetic eye screening globally.
